# Pancreatic and biliary safety signals of GLP-1 receptor agonists and tirzepatide: a disproportionality analysis of FAERS reports

**DOI:** 10.3389/fendo.2026.1941948

**Published:** 2026-09-28

**Authors:** Yuanbin Wu, Ke Li, Yuhong Wang, Zike Chen, Min Shi, Jiawei Zhao

**Affiliations:** 1 Department of Emergency Medicine, The Seventh Medical Center, Chinese PLA General Hospital, Beijing, China; 2 Department of Gastroenterology, Tongren Hospital, Shanghai Jiao Tong University School of Medicine, Shanghai, China

**Keywords:** biliary adverse events, FAERS, glucagon-like peptide-1 receptor agonists, pancreatic adverse events, pharmacovigilance, tirzepatide

## Abstract

**Background:**

The use of glucagon-like peptide-1 receptor agonists (GLP-1RAs) and tirzepatide is increasing rapidly worldwide, and the pancreatic and biliary safety of these drugs has been a concern. This study was based on the US Food and Drug Administration Adverse Event Reporting System (FAERS) to evaluate the pancreatic and biliary adverse event reporting signals of liraglutide, semaglutide, tirzepatide and dulaglutide.

**Objectives:**

To describe the reporting patterns of pancreatic and biliary adverse events for liraglutide, semaglutide, dulaglutide, and tirzepatide, and to identify signals in both organ systems within a single framework.

**Design:**

Retrospective disproportionality analysis of individual case safety reports.

**Methods:**

FAERS data from Q3–2009 through Q3–2025 were extracted, and deduplicated ICSRs were used as the unit of analysis. Reporting odds ratios (RORs) and information components (ICs) were computed at composite and Preferred Term levels; signals required the lower 95% confidence bound for the ROR to exceed 1 and IC025 to exceed 0. Metformin and sodium-glucose cotransporter-2 inhibitors were included as contextual benchmarks. Analyses were stratified by brand (diabetes/obesity indication), sex, age, and reporter type, and time to onset was examined.

**Results:**

A disproportionality signal for pancreatic adverse events was observed for all four study drugs, but the control drugs metformin and SGLT2i also showed positive signals. For biliary events, all four study drugs showed signals, whereas the two control drugs showed no signals. After stratification by brand, the pancreatic signal remained for brands with diabetes indications, while the signal was weaker for brands with obesity indications. After excluding the cases of biliary tract co-reporting, the pancreatic signal still existed. Median time to onset ranged from 85.5 to 136 days for pancreatic events and from 146.5 to 320 days for biliary events.

**Conclusion:**

Both GLP-1RAs and tirzepatide are associated with disproportionate reporting of pancreatic and biliary adverse events in FAERS. Signals are influenced by indication, reporting behavior, and time effects and cannot be used to estimate incidence or prove causality. These signals suggest the need for further validation in real-world cohort studies.

## Introduction

Glucagon-like peptide-1 receptor agonists (GLP-1RAs) and GIP/GLP-1 dual receptor agonists have rapidly changed the treatment landscape of type 2 diabetes mellitus (T2DM) and obesity, which continue to cause an increasing health burden worldwide (1). Drugs such as liraglutide, semaglutide, dulaglutide and tirzepatide have been widely used in clinical practice due to their long-lasting glycemic control, significant weight loss and proven cardiovascular benefits (2–5). As indications have expanded from diabetes to obesity management and cardiovascular risk reduction, exposure to GLP-1RAs and GIP/GLP-1 dual-receptor agonists has increased at an unprecedented rate. This trend underscores the need for rigorous and careful evaluation of their long-term safety.

Although GLP-1RAs and dual GIP/GLP-1 receptor agonists generally have favorable benefit-risk profiles, accumulating post-marketing evidence suggests that their potential gastrointestinal and hepatopancreatobiliary side effects are still worthy of attention. Pancreatic adverse events, especially acute pancreatitis, have long been a focus of regulatory attention. In recent years, biliary diseases such as cholelithiasis and cholecystitis have gradually become clinically significant safety signals. These events may be related to GLP-1RA-related changes in gallbladder motility, changes in bile composition, and rapid weight loss. Pancreatic diseases and biliary tract diseases are closely related in anatomical structure and biological mechanism, but the two diseases are often discussed separately in previous studies, which may limit the understanding of the common or different risk patterns related to GLP-1RAs and GIP/GLP-1 dual receptor agonist exposure (6, 7).

The existing evidence base remains inconclusive. Randomized controlled trials are often underpowered to detect rare but serious organ-specific adverse events and commonly exclude patients with pre-existing pancreatic or biliary disease (2–5). Observational studies and meta-analyses have produced inconsistent results and are frequently restricted to individual drugs, isolated outcomes, or aggregated adverse event categories (8–10). Importantly, relatively few studies have examined adverse events at the Preferred Term (PT) level, where clinically meaningful distinctions such as acute versus necrotizing pancreatitis or cholelithiasis versus cholecystitis can be identified (11–13). As a result, an integrated evaluation of pancreatic and biliary adverse events remains lacking. Recent FAERS-based pharmacovigilance studies across diverse therapeutic settings have further shown that disproportionality analyses can identify both known and unexpected adverse-event signals at the PT level and can provide useful post-marketing safety insights (14–17).

We therefore evaluated pancreatic and biliary adverse-event reporting for four widely used incretin-based therapies in FAERS. The analysis combined composite and Preferred Term disproportionality estimates, contextual benchmark drugs, stratification by demographic and reporter characteristics, and time-to-onset modeling. The objectives were to characterize drug-specific reporting patterns, compare pancreatic and biliary phenotypes within one framework, and generate clinically relevant hypotheses for further longitudinal study.

## Methods

### Study design and reporting criteria

We conducted a retrospective disproportionality analysis of individual case safety reports. Reporting was guided by the Reporting of A Disproportionality analysis for Drug Safety signal detection using individual case safety reports in PharmacoVigilance (READUS-PV) statement; completed READUS-PV checklists are provided as supplementary submission files (18). Because this secondary study used publicly available, de-identified data, ethics committee approval and informed consent were not required.

### Data sources

Individual case safety reports (ICSRs) are derived from quarterly files of the US Food and Drug Administration Adverse Event Reporting System (FAERS) and cover the third quarter of 2009 through the third quarter of 2025. A total of 21,645,384 raw records were retrieved, of which 3,363,487 duplicate records were removed using a global cross-quarter deduplication scheme (the highest PRIMARYID was retained for each CASEID; for the AERS format [2009–2012], the highest ISR per CASE was retained), yielding 18,281,897 unique ICSRs. Data cleaning, drug identification, and statistical analysis were performed with Python 3.11, and the analysis code is provided as **Supplementary Material**.

### Data cleaning and deduplication

All quarterly FAERS data sets were imported and integrated for this study prior to formal analysis. Duplicate reports were removed according to the FDA recommended principle, that is, the report with the highest PRIMARYID in the same CASEID was retained as the most recent version of the case. Reports lacking information on valid medications or adverse events were excluded. Drug names were standardized by uniformly mapping trade names, generic names, and common spelling variants to the corresponding active ingredient. To improve signal specificity, the primary analysis included only reports in which the study drug was flagged as the primary suspect drug. The unit of analysis was the individual case safety report (ICSR) after removing duplicates. In the analysis of composite end points, when more than one target event was included in the same ICSR, it was counted only once.

### Study drugs

The study evaluated liraglutide, semaglutide, and dulaglutide (GLP-1RAs), and tirzepatide (a dual glucose-dependent insulinotropic polypeptide/GLP-1 receptor agonist). Each active ingredient was analyzed separately.

### Adverse event identification

Pancreatic and biliary adverse events were identified using Preferred Terms (PTs) from the Medical Dictionary for Regulatory Activities (MedDRA) version 28.1; the PTs used and their MedDRA codes are provided in **Supplementary Table S1**. The pancreatic composite endpoint comprised Pancreatitis acute (10033647), Pancreatitis (10033645) and Pancreatitis necrotizing (10033654). The biliary composite endpoint comprised Cholecystitis (10008612), acute cholecystitis (10008614), chronic cholecystitis (10008617), Cholecystitis infective (10062631), Cholelithiasis (10008629), Biliary colic (10004663) and Bile duct stone (10004637). These pancreatic PTs were selected as the primary clinical diagnostic outcomes, since alcoholic and autoimmune pancreatitis represent competing etiologies, and isolated enzyme elevations do not correspond to clinically diagnosed pancreatitis. When more than one target PT was included in the same report, it was counted only once. As a sensitivity analysis, we repeated the analysis using the narrow component of the Acute pancreatitis SMQ (20000022) and the Gallstone related disorders SMQ (20000127), and analyzed reports of cholecystectomy (10008611) separately.

### Descriptive analysis

Descriptive analyses were conducted to summarize the clinical and demographic characteristics of reports involving pancreatic and biliary adverse events associated with each GLP-1 RA. Variables included sex, age, body weight, reporter type, reporter country, seriousness of the report, and clinical outcomes. Annual reporting trends for pancreatic and biliary adverse events were also assessed to characterize temporal patterns over the study period.

### Disproportionality analysis

Frequentist and Bayesian methods were used for the disproportionality analysis. The reporting odds ratio (ROR) and its 95% confidence interval, as well as the information component (IC) and its 95% credibility interval, were calculated from two-by-two contingency tables. Contingency tables were defined as follows: a = number of reports of the specific drug and target event, b = number of reports of the specific drug and other events, c = number of reports of other drugs and the target event, d = number of reports of other drugs and other events. The background population (N) was all deduplicated individual case safety reports (N = 18,281,897) in the locked dataset, and the same background population was used for all drugs. The signal criterion was a lower limit of the 95% confidence interval of the ROR > 1 and IC025 > 0. When any cell in a contingency table was 0, 0.5 was added to each cell. The analysis used deduplicated individual case safety reports as the unit of analysis, and an ICSR containing multiple target events was counted only once. The primary analysis included only the primary suspect (PS) role; sensitivity analyses including the secondary suspect role are reported separately. Analyses covered composite endpoints and individual Preferred Terms (PTs). The structure of the two-by-two contingency table is shown in **Supplementary Table S2**, the underlying cell counts for each drug and outcome in **Supplementary Table S3**, and the formulae and signal-detection criteria in **Supplementary Table S4** (19). PT-level analyses can improve the granularity and clinical interpretability of signal detection (17).

### Contextual benchmark analysis

Metformin and sodium-glucose cotransporter-2 inhibitors (empagliflozin, dapagliflozin, canagliflozin, and ertugliflozin) were analyzed as contextual benchmark drugs. Their disproportionality estimates were calculated independently against the same FAERS reporting background used for the study drugs. They were not used as denominators in pairwise drug-versus-drug comparisons.

### Stratified and sensitivity analyses

Subgroup disproportionality analyses were performed by sex, age group, and reporter type. Reporters were classified as healthcare professionals or consumers. ROR and IC025 were recalculated within each subgroup according to the prespecified signal criteria, and subgroup estimates were interpreted descriptively without formal interaction testing. To evaluate the robustness of the results, a series of sensitivity analyses were conducted, including: extending the primary analysis to primary and secondary suspect roles; restricting the analysis to a common post-marketing period (from 2022) to balance differences in market entry and reporting environment; restricting to reports recorded in the INDI table as type 2 diabetes or obesity indications; stratifying by brand, using brand as a proxy for indication (e.g., Mounjaro and Ozempic for diabetes, Zepbound and Wegovy for obesity); re-running the analyses using the MedDRA standard SMQs (the narrow component of the Acute pancreatitis SMQ 20000022 and the Gallstone related disorders SMQ 20000127); analyzing cholecystectomy reports separately; evaluating co-reporting of pancreatic and biliary events and recalculating the pancreatic signal after excluding reports with biliary events; and calculating the annual reporting proportion per drug (events divided by total reports for that drug in that year) to adjust for variation in reporting volume.

### Time-to-onset analysis

Time to onset was defined as the interval between the recorded therapy start date and the adverse-event onset date. Only reports with complete and chronologically plausible dates were included. Cumulative distribution functions described the temporal patterns, and distributions were compared using the Kruskal-Wallis test. Weibull models characterized the reporting-time patterns: a shape parameter β < 1 indicated an early-failure pattern, β approximately 1 a random-failure pattern, and β > 1 a wear-out pattern (20). Similar time-to-onset methods have been used in recent FAERS studies (14, 15, 17).

### Statistics

All data management and statistical analyses were performed using Python 3.11. Disproportionality analyses used a purpose-built two-by-two contingency-table pipeline for ROR/IC computation. Drug identification and PT matching were based on the dictionaries provided in the **Supplementary Material**. The Kruskal-Wallis test and Weibull models were implemented in SciPy. The full analysis code and dependency versions are provided in the **Supplementary Material**. All statistical tests were two-sided, and statistical significance was assessed according to prespecified criteria.

## Results

### Data overview of pancreatic and biliary adverse events reported in FAERS

During the study period, 21,645,384 raw FAERS records were retrieved, and after the global deduplication scheme described in the Methods, 18,281,897 unique individual case safety reports (ICSRs) were included in the analysis. Among primary-suspect reports, pancreatic events were reported in 2,891 with liraglutide, 1,579 with semaglutide, 1,093 with tirzepatide, and 1,556 with dulaglutide. The corresponding numbers of biliary events were 528, 931, 562, and 247 (**Figure 1**). Demographic information is provided in **Supplementary Table S5**.

**Figure 1:**
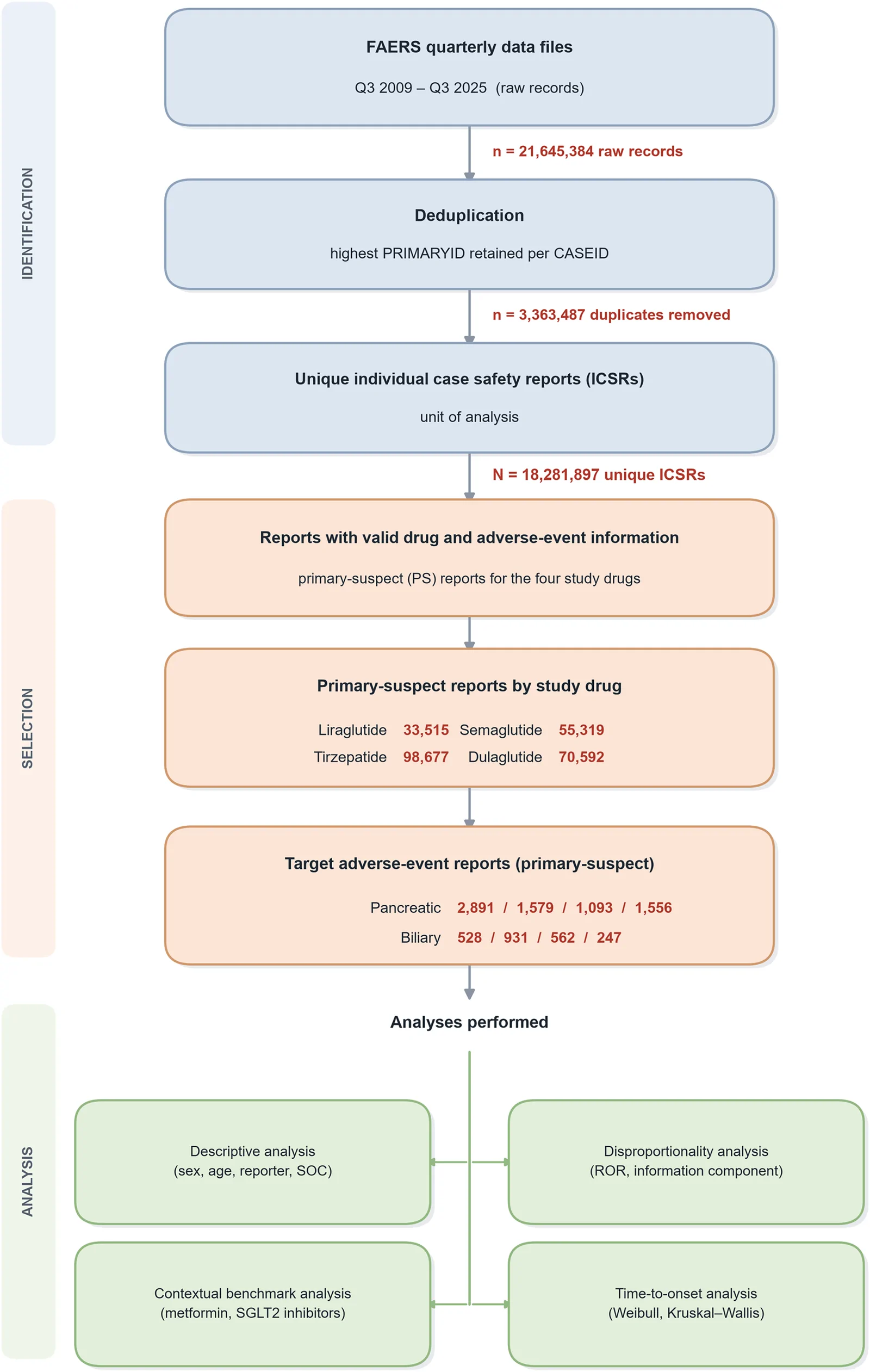
Flowchart of report selection and analysis. FAERS reports from Q3–2009 through Q3–2025 were retrieved (21,645,384 raw records) and deduplicated using CASEID and PRIMARYID to 18,281,897 unique ICSRs. Reports with valid drug and adverse-event information were retained, and primary-suspect reports for liraglutide, semaglutide, tirzepatide, and dulaglutide were identified. Prespecified pancreatic and biliary Preferred Terms were evaluated using descriptive, disproportionality, contextual benchmark, subgroup, and time-to-onset analyses.

### Distribution across System Organ Classes

There was significant variation in the distribution of adverse events across system organ classes (SOC) for the four study drugs, with gastrointestinal, neurological, and systemic disorders reported most frequently across all drugs (**Supplementary Table S6**).

### Descriptive characteristics of adverse-event reports

Women accounted for 49.6%-66.1% of reported pancreatic events across study drugs. Among reports with age recorded, 45–64 years was the most frequent group (50.7%-60.1%). Hospitalization was reported in 42.5%-49.7% of pancreatic-event reports and 53.6%-63.6% of biliary-event reports (**Table 1**; **Supplementary Table S7**). These proportions describe the submitted reports and do not represent population risk.

**Table 1:** Clinical and demographic characteristics of pancreatic adverse events associated with Liraglutide, Semaglutide, Tirzepatide and Dulaglutide.

Characteristic	Reports of pancreatic adverse events			
	Liraglutide n = 2891, (%)	Semaglutide n = 1579, (%)	Tirzepatide n = 1093, (%)	Dulaglutide n = 1556, (%)
Sex				
Female	1434 (56.5)	812 (55.0)	536 (66.1)	626 (49.6)
Male	1104 (43.5)	665 (45.0)	275 (33.9)	635 (50.4)
Age (year)				
<18	6 (0.3)	5 (0.5)	0 (0.0)	2 (0.3)
18-44	272 (15.1)	141 (15.0)	160 (30.2)	61 (8.5)
45-64	1084 (60.1)	519 (55.2)	268 (50.7)	414 (58.0)
≥65	442 (24.5)	276 (29.3)	101 (19.1)	237 (33.2)
Weight (kg)				
≤80	202 (16.3)	123 (27.0)	56 (26.3)	58 (21.0)
80-100	411 (33.1)	148 (32.5)	80 (37.6)	100 (36.2)
≥100	630 (50.7)	185 (40.6)	77 (36.2)	118 (42.8)
Reporter type				
Consumer	497 (17.6)	444 (28.3)	994 (91.1)	1200 (77.5)
Health Professional	645 (22.8)	369 (23.5)	45 (4.1)	138 (8.9)
Pharmacist	130 (4.6)	141 (9.0)	9 (0.8)	61 (3.9)
Physician	1555 (55.0)	614 (39.2)	43 (3.9)	149 (9.6)
Outcome				
Death	52 (1.8)	58 (3.7)	26 (2.4)	33 (2.1)
Disability	23 (0.8)	28 (1.8)	17 (1.6)	12 (0.8)
Hospitalization	1417 (49.0)	785 (49.7)	493 (45.1)	661 (42.5)
Life-Threatening	72 (2.5)	82 (5.2)	92 (8.4)	48 (3.1)
Other	1327 (45.9)	625 (39.6)	465 (42.5)	801 (51.5)

### Annual reporting trends

The normalized report proportions (events per drug per year divided by total reports for that drug in that year) showed that pancreatic-event proportions decreased over time for liraglutide and semaglutide and were relatively stable for tirzepatide (**Figure 2A**). The normalized biliary-event proportions also decreased for liraglutide and semaglutide and were relatively stable for tirzepatide and dulaglutide (**Figure 2B**). Values for 2025 include only Q1-Q3 and are not directly comparable with complete years.

**Figure 2:**
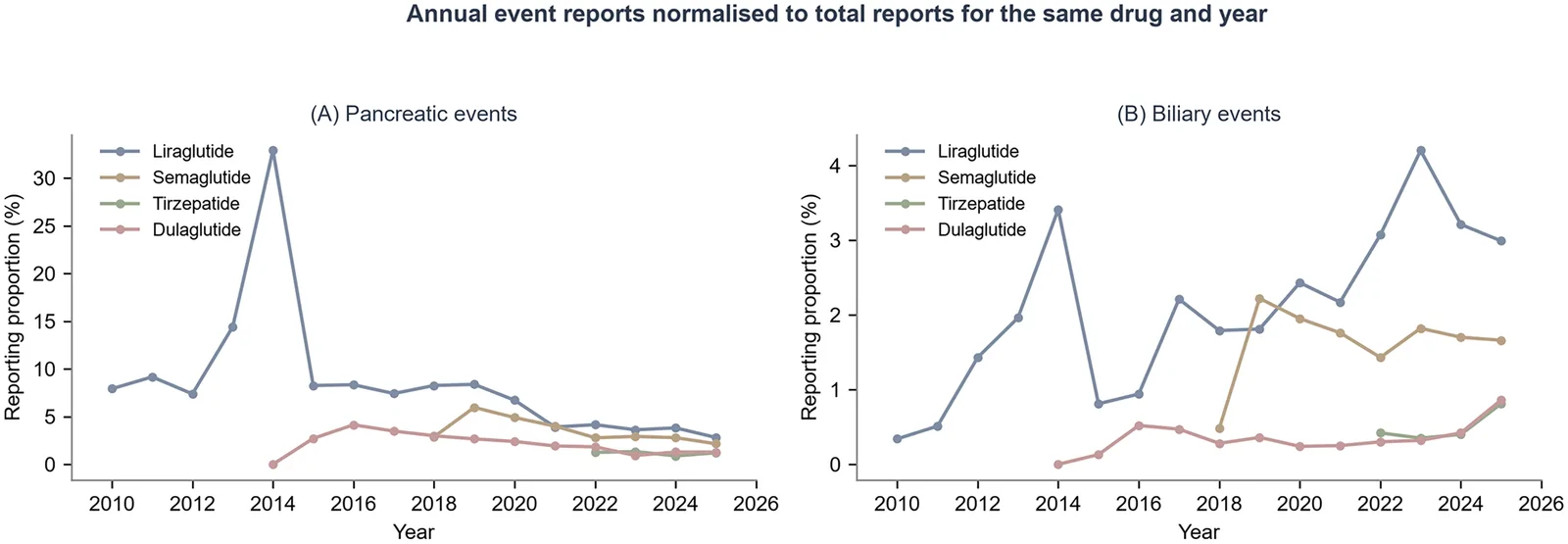
Annual numbers of pancreatic **(A)** and biliary **(B)** adverse-event reports associated with the study drugs from 2010 to 2025. Data for 2025 include Q1-Q3 only.

### Disproportionality analysis of pancreatic and biliary adverse events

All four study drugs met the prespecified signal criteria: liraglutide (ROR 28.53, 95%CI 27.44-29.66; IC025 4.60), semaglutide (ROR 8.68; IC025 2.98), dulaglutide (ROR 6.65; IC025 2.61), tirzepatide (ROR 3.27; IC025 1.60). Control estimates were ROR 4.20 (IC025 1.95) for metformin and ROR 3.78 (IC025 1.81) for SGLT-2 inhibitors (**Table 2**). Each estimate was based on the same FAERS reporting background, and these values are not pairwise comparative effect estimates.

**Table 2:** Disproportionality estimates for pancreatic adverse events, including contextual benchmark drugs.

Drug	Case (n)	ROR	95% CI	IC	IC025-IC975
Metformin	954	4.20	3.94-4.48	2.04	1.95-2.13
SGLT2i	1136	3.78	3.57-4.01	1.89	1.81-1.97
Dulaglutide	1556	6.65	6.32-7.00	2.68	2.61-2.75
Liraglutide	2891	28.53	27.44-29.66	4.64	4.60-4.69
Semaglutide	1579	8.68	8.25-9.13	3.05	2.98-3.12
Tirzepatide	1093	3.27	3.08-3.48	1.68	1.60-1.77

Biliary-event signals were smaller. Liraglutide (ROR 6.06; IC025 2.45), semaglutide (ROR 6.52; IC025 2.57), tirzepatide (ROR 2.16; IC025 0.98), and dulaglutide (ROR 1.32; IC025 0.22) met the prespecified criteria. Metformin (ROR 1.02; IC025-0.18) and SGLT-2 inhibitors (ROR 0.74; IC025 -0.64) did not reach a signal (**Table 3**). These independently calculated reporting estimates do not establish relative risk between drugs.

**Table 3:** Disproportionality estimates for biliary adverse events, including contextual benchmark drugs.

Drug	Case (n)	ROR	95% CI	IC	IC025-IC975
Metformin	182	1.02	0.88-1.18	0.03	-0.18-0.24
SGLT2i	176	0.74	0.64-0.86	-0.42	-0.64--0.21
Dulaglutide	247	1.32	1.16-1.49	0.40	0.22-0.58
Liraglutide	528	6.06	5.55-6.60	2.57	2.45-2.69
Semaglutide	931	6.52	6.11-6.96	2.66	2.57-2.75
Tirzepatide	562	2.16	1.99-2.35	1.10	0.98-1.22

### Preferred Term-level analyses

At the Preferred Term level, pancreatitis and acute pancreatitis were the prominent pancreatic terms, with estimates varying across drugs and liraglutide among the highest for several terms (**Supplementary Table S8**). Cholelithiasis and biliary colic were prominent biliary terms, particularly in liraglutide and semaglutide reports (**Supplementary Table S9**).

### Subgroup disproportionality analyses

Subgroup estimates were descriptive. The lower 95% bounds for pancreatic events by subgroup are shown in **Figure 3**: liraglutide showed a large estimate in healthcare-professional reports (ROR 51.8) and a lower estimate in consumer reports (ROR 11.6). Estimates for the four drugs across male, female, 18-44, 45-64, and ≥65 years subgroups are shown in **Figure 3** (pancreatic) and **Figure 4** (biliary). Because interaction tests were not performed, these differences should not be interpreted as evidence of subgroup-specific risk.

**Figure 3:**
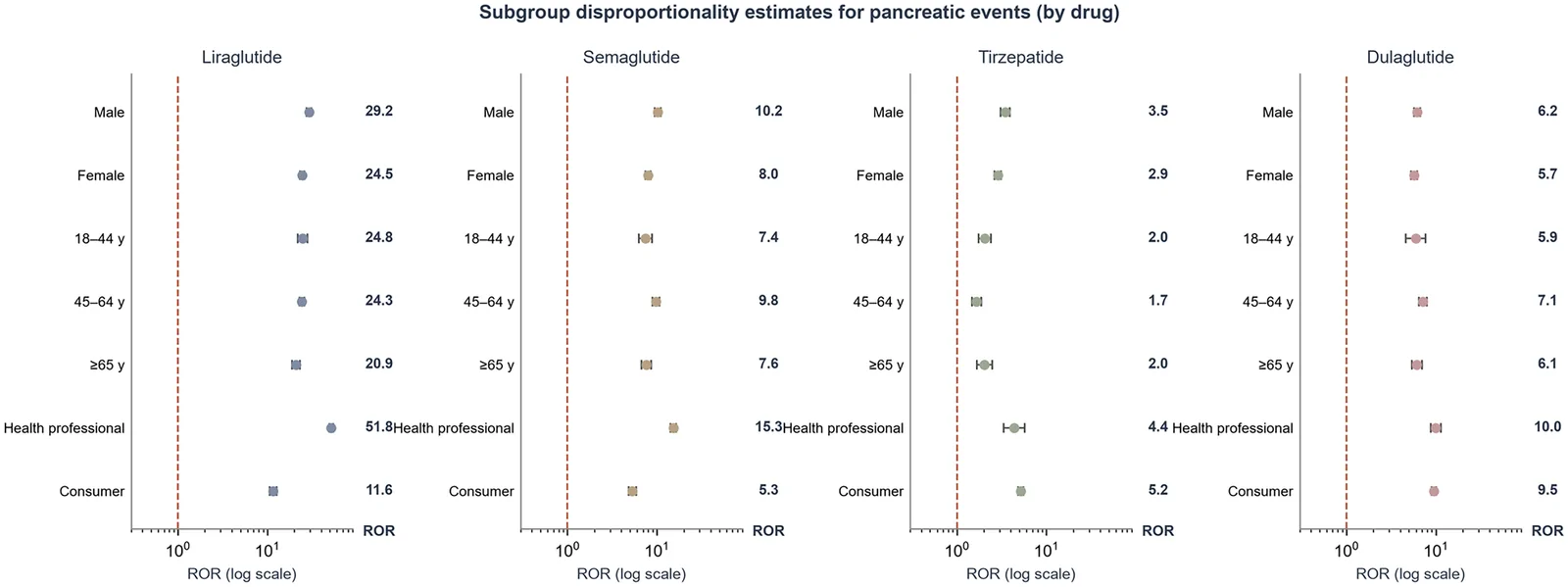
Subgroup disproportionality estimates for pancreatic adverse-event reports by sex, age, and reporter type. ROR, reporting odds ratio; CI, confidence interval; IC025, lower bound of the 95% credibility interval for the information component.

**Figure 4:**
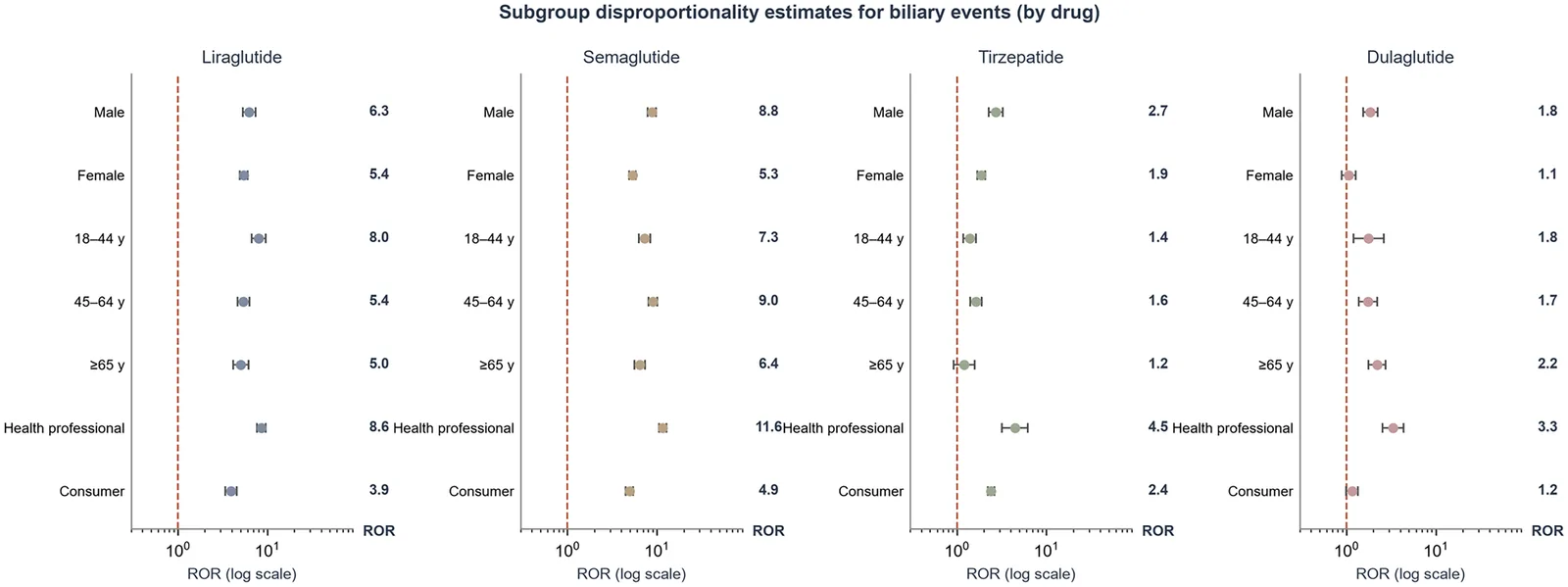
Subgroup disproportionality estimates for biliary adverse-event reports by sex, age, and reporter type. ROR, reporting odds ratio; CI, confidence interval; IC025, lower bound of the 95% credibility interval for the information component.

### Time-to-onset analyses

Median time to onset for pancreatic events was 101 days for liraglutide (IQR 32-286), 136 days for semaglutide (IQR 42-413), 93 days for tirzepatide (IQR 31-228), and 85.5 days for dulaglutide (IQR 27-275), with a significant difference in distribution (Kruskal-Wallis p=0.0069) (**Table 4**; **Figure 5A**). Median time to onset for biliary events was 160 days for liraglutide (IQR 54-404), 320 days for semaglutide (IQR 123-769), 146.5 days for tirzepatide (IQR 49-311), and 232 days for dulaglutide (IQR 69-940), with a significant difference (p < 0.001) (**Table 5**; **Figure 5B**). The Weibull shape parameters were below 1 for all four drugs for both pancreatic and biliary events, consistent with an early-onset reporting pattern (**Tables 4**, **5**).

**Table 4:** Time-to-onset analysis of study drug-related signals of pancreatic adverse events using the Weibull distribution test.

Drug	Time-to-onset (days)			Weibull distribution				Failure type
				Scale parameter		Shape parameter		
	N	Median (IQR)	Min-Max	α	95% CI	β	95% CI	
Liraglutide	953	101 (32, 286)	1-4623	205.99	185.39-226.58	0.67	0.64-0.70	Early failure
Semaglutide	361	136 (42, 413)	1-4018	297.87	244.11-351.62	0.60	0.56-0.65	Early failure
Tirzepatide	196	93 (31, 228)	1-4687	189.53	143.18-235.87	0.61	0.55-0.67	Early failure
Dulaglutide	240	85.5 (27, 275)	1-3919	186.82	147.15-226.50	0.63	0.57-0.69	Early failure

**Figure 5:**
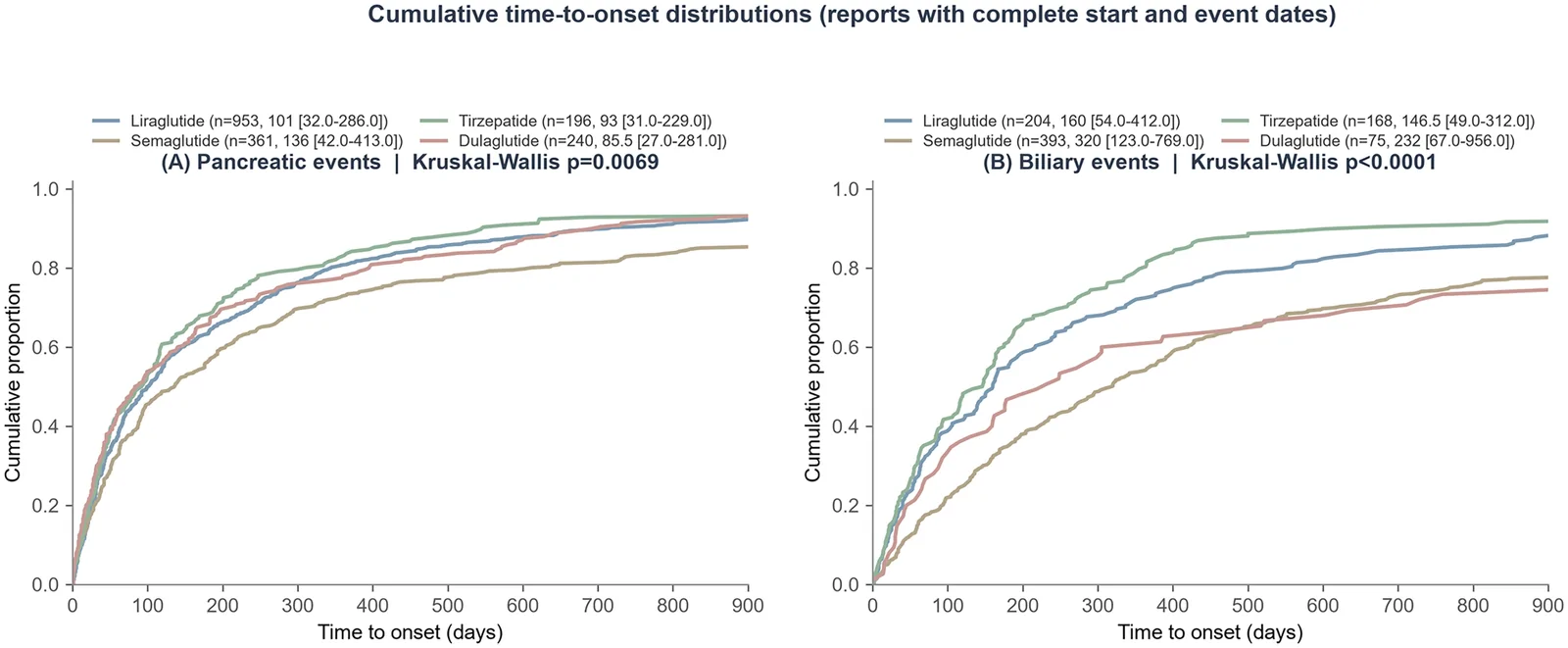
Cumulative time-to-onset distributions for pancreatic **(A)** and biliary **(B)** adverse-event reports associated with dulaglutide, liraglutide, semaglutide, and tirzepatide. Pancreatic distributions differed significantly across drugs (Kruskal-Wallis p = 0.0069), as did biliary distributions (p < 0.001).

**Table 5:** Time-to-onset analysis of study drugs-related signals of biliary adverse events using the Weibull distribution test.

Drug	Time-to-onset (days)			Weibull distribution				Failure type
				Scale parameter		Shape parameter		
	N	Median (IQR)	Min-Max	α	95% CI	β	95% CI	
Liraglutide	204	160 (54, 404)	2-4623	296.10	236.33-355.86	0.72	0.65-0.79	Early failure
Semaglutide	393	320 (123, 769)	1-4899	577.47	497.96-656.97	0.76	0.70-0.82	Early failure
Tirzepatide	168	146.5 (49, 311)	1-4687	253.35	191.69-315.02	0.66	0.59-0.73	Early failure
Dulaglutide	75	232 (69, 940)	1-2588	466.27	317.00-615.55	0.75	0.61-0.88	Early failure

### Sensitivity analyses

After stratification by brand, the pancreatic signal remained for diabetes-indication brands (e.g., tirzepatide Mounjaro, ROR 4.06; semaglutide Ozempic/Rybelsus, ROR 9.32), while the signal was weaker for obesity-indication brands (e.g., tirzepatide Zepbound, ROR 1.54; semaglutide Wegovy, ROR 5.68).

The number of co-reported ICSRs for pancreatic and biliary events was 155 for liraglutide, 118 for semaglutide, 98 for tirzepatide, and 62 for dulaglutide. After excluding reports with biliary events, the pancreatic signal of each drug persisted (liraglutide ROR 28.50, semaglutide 8.49, tirzepatide 3.16, dulaglutide 6.77) (**Supplementary Table S10**). The most common co-reported pancreatic and biliary pair was pancreatitis with cholelithiasis for each drug (**Supplementary Table S11**).

Sensitivity analysis using the narrow component of the Acute pancreatitis SMQ (20000022) and the Gallstone related disorders SMQ (20000127) showed that the core signal remained robust: the RORs for liraglutide, semaglutide, tirzepatide, and dulaglutide under the narrow component of the Acute pancreatitis SMQ were 28.00, 8.56, 3.22, and 6.54, respectively; under the narrow component of the Gallstone SMQ, they were 7.02, 6.70, 2.48, and 1.45. Of note, metformin showed a weak signal (ROR 1.23, IC025 0.06) in the narrow component of the Gallstone SMQ, while SGLT-2 inhibitors showed no signal (**Supplementary Table S12**).

## Discussion

This FAERS pharmacovigilance study evaluated pancreatic and biliary adverse event reports for three GLP-1 receptor agonists and tirzepatide within a single framework. Composite endpoints were combined with Preferred Term disproportionality estimates, benchmark comparator drugs, subgroup reporting patterns, and time-to-onset analysis. The pancreatic reporting signal was greater than the biliary signal, with estimates varying by drug and clinical phenotype (21–23).

All four study drugs met the pancreatic event signal criteria. It should be noted that pancreatic signals were also observed for the benchmark drugs metformin and SGLT-2 inhibitors, suggesting that this pancreatic reporting pattern is not unique to the GLP-1 drug class. Benchmark estimates provide a background reference within the same database but do not control for differences in indication, exposure, or reporting behavior and should not be interpreted as pairwise comparisons between drugs (24, 25). Although this study did not demonstrate a unique pancreatic reporting pattern for GLP-1 drugs or tirzepatide, after stratification by brand, the pancreatic signal remained for diabetes-indication brands (e.g., Mounjaro, Ozempic), while the signal was weaker for obesity-indication brands (e.g., Zepbound). When restricted to patients with type 2 diabetes, the pancreatic signal of most drugs disappeared. These results suggest that signals may be influenced by indication, reporting population, and reporting behavior rather than by drug effects alone.

Reporting behavior may itself be shaped by external factors. Media attention and regulatory alerts can induce stimulated reporting, as illustrated by the peak of liraglutide reports in 2013–2014 and the predominance of consumer reports for tirzepatide pancreatic events, which may affect signal detection and interpretation.

Biliary signals were smaller and were greatest for liraglutide and semaglutide (26, 27). The difference between pancreatic and biliary estimates is a reporting pattern, not evidence of organ-specific susceptibility (2–4, 28).

At the Preferred Term level, the analyses clarified the clinical composition of the composite endpoints. Pancreatitis and acute pancreatitis were prominent pancreatic terms, and estimates for liraglutide were generally large; cholelithiasis and biliary colic were prominent biliary terms. Cholecystitis-related estimates were smaller or inconsistent for some drugs. PT-level analyses can reveal heterogeneity that is masked by broader outcome groupings, but they remain subject to reporter-selected terminology and coding variation (22, 29).

The joint assessment of pancreatic and biliary reports has clinical value because these organ systems are anatomically and functionally related and their disorders can have overlapping manifestations (30, 31). We found that the proportion of co-reporting of pancreatic and biliary events was low (3%-9%), and pancreatic signals remained significant for each drug after excluding reports containing biliary events, indicating that pancreatic signals were independent of biliary co-reporting. However, FAERS cannot determine whether pancreatic events are secondary to biliary tract disease or distinguish direct drug effects from pathways involving weight loss or gallbladder motility. Thus, the dual-organ framework should be viewed as a descriptive tool for hypothesis generation rather than as a means of mechanism attribution (32, 33).

The time-to-onset analysis showed that the median reporting time of pancreatic events was earlier than that of biliary events for some drugs, and most drug-event combinations showed an early-onset Weibull pattern (34). These patterns may help define periods of clinical vigilance, but incomplete dates and selective reporting limit inference. In particular, the temporal distributions do not prove acute pharmacological effects, cumulative toxicity, or causality.

The greater signal reported by healthcare professionals than by consumers may reflect differences in diagnostic certainty, event seriousness, or reporting practices. Sex and age differences were smaller. Given the absence of formal interaction tests and exposure denominators, subgroup results should not be interpreted as evidence of differences in susceptibility or broad population extrapolation.

The findings should be interpreted in the context of available evidence. Major randomized trials (LEADER, SUSTAIN-6, REWIND, and SURPASS) have generally not shown significant increases in GLP-1RA-related pancreatitis, but these trials were not designed primarily to detect rare pancreatic outcomes and may have limited power for specific clinical phenotypes (2–5). Biliary tract events have been observed in obesity trials, particularly with high doses of semaglutide and liraglutide, and may be influenced by rapid weight loss and altered gallbladder motility. Furthermore, real-world cohort evidence from a large target-trial simulation comparing GLP-1RA and tirzepatide initiation showed a reduction in all-cause mortality but a modest increase in the risk of acute pancreatitis (HR 1.06) (35), with the risk concentrated in the first 6 months of treatment, suggesting that the FAERS signal is consistent with real-world epidemiologic evidence but of small magnitude, and that early monitoring is clinically relevant.

Clinically, these findings do not alter the established benefits of incretin therapies for glycemic control, weight management, and cardiovascular outcomes. They support attention to pancreatic symptoms and biliary complications during treatment, especially in patients with relevant histories. Decisions should remain individualized, as FAERS signals do not quantify absolute risk.

## Limitations

There are several limitations to this study that need to be considered with caution. As a spontaneous reporting system, FAERS is inevitably subject to underreporting, selective reporting, and provocative reporting induced by media attention and regulatory alerts, which may alter adverse-event reporting patterns over time. Estimates of incidence or absolute risk cannot be made because of a lack of denominator data. Therefore, all results should be interpreted strictly as a disproportionality reporting signal and not as a direct measure of risk magnitude or causality. Clinical details, such as dose, exposure duration, imaging findings, and diagnostic confirmation, are often incomplete, potentially leading to misclassification of exposures and outcomes.

Confounding by indication remains important because obesity, type 2 diabetes, and related metabolic conditions are themselves associated with pancreatic and biliary disease. Contextual benchmark estimates do not eliminate systematic differences between drug-report groups, and residual confounding cannot be excluded. Direct comparison of disproportionality metrics across drugs also requires caution because exposure and reporting patterns differ.

Analyses restricted to primary suspect drugs may improve specificity but reduce sensitivity, potentially leading to underestimation of signals. In addition, PT-level analyses rely on reporter-selected terminology and MedDRA coding, which may vary and introduce heterogeneity. Time-to-onset analyses were limited to cases with complete date information, which may introduce selection bias and affect the interpretation of temporal patterns.

Finally, the close anatomical and clinical relationship between pancreatic and biliary diseases introduces interpretative complexity, as some pancreatic events may be secondary to biliary pathology and cannot be disentangled using FAERS data alone. For newer agents such as tirzepatide, shorter post-marketing exposure and potentially higher reporting rates may have influenced signal detection. Therefore, as with other FAERS-based disproportionality analyses, these findings should be considered hypothesis-generating rather than causal and warrant confirmation in well-designed longitudinal and mechanistic studies (36).

## Conclusion

In summary, FAERS showed drug-specific disproportionate reporting patterns for pancreatic and, to a lesser extent, biliary adverse events. FAERS data suggest disproportionate reporting signals for pancreatic and biliary events for all four incretin therapies, as well as similar pancreatic signals for the comparator drugs metformin and SGLT2 inhibitors. Thus, this analysis did not demonstrate a unique pancreatic reporting pattern for GLP-1 receptor agonists or tirzepatide.

## Data Availability

Publicly available datasets were analyzed in this study. This data can be found here: The existing datasets analyzed in this study is publicly available from the FDA Adverse Event Reporting System (FAERS) Quarterly Data Extract Files: https://fis.fda.gov/extensions/FPD-QDE-FAERS/FPD-QDE-FAERS.html. No accession number is applicable.
