# Supplementary material for: Pancreatic and biliary safety signals of GLP-1 receptor agonists and tirzepatide: a disproportionality analysis of FAERS reports
Source: Front Endocrinol (Lausanne). 2026 Sep 28;17:1941948. doi: 10.3389/fendo.2026.1941948 (PMC13654742; doi:10.3389/fendo.2026.1941948)
Supplement: Supplementary file 1 [file Table1.docx]

**Supplementary Tables S1-S12**

**Pancreatic and biliary safety signals of GLP-1 receptor agonists and tirzepatide: a disproportionality analysis of FAERS reports**

**Table S1. List of pancreatic and biliary adverse reactions included in this study.**

**Preferred terms for pancreatic adverse events**

Pancreatitis acute (10033647)

Pancreatitis (10033645)

Pancreatitis necrotizing (10033654)

**Preferred terms for biliary adverse events**

Cholecystitis (10008612)

Cholecystitis chronic (10008617)

Cholecystitis acute (10008614)

Cholelithiasis (10008629)

Cholecystitis infective (10062631)

Biliary colic (10004663)

Bile duct stone (10004637)

Pancreatic and biliary adverse events were defined using selected Medical Dictionary for Regulatory Activities (MedDRA) Preferred Terms (PTs) recorded in the FAERS database. PTs represent standardized medical concepts for adverse reactions, including symptoms, clinical signs, and disease diagnoses, and were used as the analytical unit in this study. The selected PTs were grouped into pancreatic and biliary categories to construct composite adverse event endpoints for subsequent disproportionality analyses.

**Table S2. Structure of the two-by-two contingency table for disproportionality analysis. Cells a, b, c, and d denote the report counts in each cell of the contingency table.**

| **Item** | **Target adverse events reported** | **Other adverse events reported** | **Total** |
| --- | --- | --- | --- |
| **Target drugs** | a | b | a + b |
| **Other drugs** | c | d | c + d |
| **Total** | a + c | b + d | a + b + c + d |

**Table S3. Two-by-two contingency table for disproportionality analysis.**

| **Drug** | **Outcome** | **a** | **b** | **c** | **d** | **ROR** | **IC025** |
| --- | --- | --- | --- | --- | --- | --- | --- |
| Liraglutide | Pancreatic composite | 2891 | 30624 | 60183 | 18188199 | 28.53 | 4.6 |
| Liraglutide | Biliary composite | 528 | 32987 | 48105 | 18200277 | 6.06 | 2.45 |
| Liraglutide | Pancreatitis acute | 640 | 32875 | 18079 | 18230303 | 19.63 | 4.11 |
| Liraglutide | Pancreatitis | 2224 | 31291 | 40991 | 18207391 | 31.57 | 4.76 |
| Liraglutide | Pancreatitis necrotizing | 36 | 33479 | 1734 | 18246648 | 11.32 | 3.03 |
| Liraglutide | Cholecystitis infective | 18 | 33497 | 2385 | 18245997 | 4.11 | 1.42 |
| Liraglutide | Cholecystitis chronic | 18 | 33497 | 6663 | 18241719 | 1.47 | -0.06 |
| Liraglutide | Cholecystitis acute | 53 | 33462 | 4970 | 18243412 | 5.81 | 2.15 |
| Liraglutide | Cholecystitis | 91 | 33424 | 9969 | 18238413 | 4.98 | 2.02 |
| Liraglutide | Cholelithiasis | 346 | 33169 | 27093 | 18221289 | 7.02 | 2.63 |
| Liraglutide | Biliary colic | 47 | 33468 | 2441 | 18245941 | 10.5 | 2.97 |
| Liraglutide | Bile duct stone | 32 | 33483 | 2680 | 18245702 | 6.51 | 2.22 |
| Semaglutide | Pancreatic composite | 1579 | 53740 | 61495 | 18165083 | 8.68 | 2.98 |
| Semaglutide | Biliary composite | 931 | 54388 | 47702 | 18178876 | 6.52 | 2.57 |
| Semaglutide | Pancreatitis acute | 257 | 55062 | 18462 | 18208116 | 4.6 | 2.01 |
| Semaglutide | Pancreatitis | 1262 | 54057 | 41953 | 18184625 | 10.12 | 3.19 |
| Semaglutide | Pancreatitis necrotizing | 75 | 55244 | 1695 | 18224883 | 14.6 | 3.5 |
| Semaglutide | Cholecystitis infective | 40 | 55279 | 2363 | 18224215 | 5.58 | 2.04 |
| Semaglutide | Cholecystitis chronic | 30 | 55289 | 6651 | 18219927 | 1.49 | 0.08 |
| Semaglutide | Cholecystitis acute | 84 | 55235 | 4939 | 18221639 | 5.61 | 2.17 |
| Semaglutide | Cholecystitis | 253 | 55066 | 9807 | 18216771 | 8.53 | 2.88 |
| Semaglutide | Cholelithiasis | 518 | 54801 | 26921 | 18199657 | 6.39 | 2.52 |
| Semaglutide | Biliary colic | 111 | 55208 | 2377 | 18224201 | 15.41 | 3.63 |
| Semaglutide | Bile duct stone | 76 | 55243 | 2636 | 18223942 | 9.51 | 2.9 |
| Tirzepatide | Pancreatic composite | 1093 | 97584 | 61981 | 18121239 | 3.27 | 1.6 |
| Tirzepatide | Biliary composite | 562 | 98115 | 48071 | 18135149 | 2.16 | 0.98 |
| Tirzepatide | Pancreatitis acute | 205 | 98472 | 18514 | 18164706 | 2.04 | 0.83 |
| Tirzepatide | Pancreatitis | 868 | 97809 | 42347 | 18140873 | 3.8 | 1.8 |
| Tirzepatide | Pancreatitis necrotizing | 25 | 98652 | 1745 | 18181475 | 2.64 | 0.86 |
| Tirzepatide | Cholecystitis infective | 28 | 98649 | 2375 | 18180845 | 2.17 | 0.61 |
| Tirzepatide | Cholecystitis chronic | 2 | 98675 | 6679 | 18176541 | 0.06 | -5.64 |
| Tirzepatide | Cholecystitis acute | 51 | 98626 | 4972 | 18178248 | 1.89 | 0.53 |
| Tirzepatide | Cholecystitis | 111 | 98566 | 9949 | 18173271 | 2.06 | 0.77 |
| Tirzepatide | Cholelithiasis | 378 | 98299 | 27061 | 18156159 | 2.58 | 1.21 |
| Tirzepatide | Biliary colic | 53 | 98624 | 2435 | 18180785 | 4.01 | 1.61 |
| Tirzepatide | Bile duct stone | 23 | 98654 | 2689 | 18180531 | 1.58 | 0.1 |
| Dulaglutide | Pancreatic composite | 1556 | 69036 | 61518 | 18149787 | 6.65 | 2.61 |
| Dulaglutide | Biliary composite | 247 | 70345 | 48386 | 18162919 | 1.32 | 0.22 |
| Dulaglutide | Pancreatitis acute | 198 | 70394 | 18521 | 18192784 | 2.76 | 1.26 |
| Dulaglutide | Pancreatitis | 1331 | 69261 | 41884 | 18169421 | 8.34 | 2.92 |
| Dulaglutide | Pancreatitis necrotizing | 32 | 70560 | 1738 | 18209567 | 4.75 | 1.76 |
| Dulaglutide | Cholecystitis infective | 11 | 70581 | 2392 | 18208913 | 1.19 | -0.52 |
| Dulaglutide | Cholecystitis chronic | 0 | 70592 | 6681 | 18204624 | 0.02 | -7.52 |
| Dulaglutide | Cholecystitis acute | 26 | 70566 | 4997 | 18206308 | 1.34 | -0.1 |
| Dulaglutide | Cholecystitis | 51 | 70541 | 10009 | 18201296 | 1.31 | 0.01 |
| Dulaglutide | Cholelithiasis | 152 | 70440 | 27287 | 18184018 | 1.44 | 0.3 |
| Dulaglutide | Biliary colic | 15 | 70577 | 2473 | 18208832 | 1.56 | -0.03 |
| Dulaglutide | Bile duct stone | 15 | 70577 | 2697 | 18208608 | 1.43 | -0.15 |
| Metformin | Pancreatic composite | 954 | 66334 | 62120 | 18152489 | 4.2 | 1.95 |
| Metformin | Biliary composite | 182 | 67106 | 48451 | 18166158 | 1.02 | -0.18 |
| Metformin | Pancreatitis acute | 550 | 66738 | 18169 | 18196440 | 8.25 | 2.88 |
| Metformin | Pancreatitis | 397 | 66891 | 42818 | 18171791 | 2.52 | 1.18 |
| Metformin | Pancreatitis necrotizing | 8 | 67280 | 1762 | 18212847 | 1.23 | -0.58 |
| Metformin | Cholecystitis infective | 7 | 67281 | 2396 | 18212213 | 0.79 | -1.27 |
| Metformin | Cholecystitis chronic | 2 | 67286 | 6679 | 18207930 | 0.08 | -5.09 |
| Metformin | Cholecystitis acute | 20 | 67268 | 5003 | 18209606 | 1.08 | -0.47 |
| Metformin | Cholecystitis | 27 | 67261 | 10033 | 18204576 | 0.73 | -0.97 |
| Metformin | Cholelithiasis | 123 | 67165 | 27316 | 18187293 | 1.22 | 0.04 |
| Metformin | Biliary colic | 13 | 67275 | 2475 | 18212134 | 1.42 | -0.21 |
| Metformin | Bile duct stone | 11 | 67277 | 2701 | 18211908 | 1.1 | -0.63 |
| SGLT2i | Pancreatic composite | 1136 | 87867 | 61938 | 18130956 | 3.78 | 1.81 |
| SGLT2i | Biliary composite | 176 | 88827 | 48457 | 18144437 | 0.74 | -0.64 |
| SGLT2i | Pancreatitis acute | 302 | 88701 | 18417 | 18174477 | 3.36 | 1.57 |
| SGLT2i | Pancreatitis | 807 | 88196 | 42408 | 18150486 | 3.92 | 1.84 |
| SGLT2i | Pancreatitis necrotizing | 41 | 88962 | 1729 | 18191165 | 4.85 | 1.83 |
| SGLT2i | Cholecystitis infective | 12 | 88991 | 2391 | 18190503 | 1.03 | -0.7 |
| SGLT2i | Cholecystitis chronic | 3 | 89000 | 6678 | 18186216 | 0.09 | -4.73 |
| SGLT2i | Cholecystitis acute | 25 | 88978 | 4998 | 18187896 | 1.02 | -0.5 |
| SGLT2i | Cholecystitis | 48 | 88955 | 10012 | 18182882 | 0.98 | -0.42 |
| SGLT2i | Cholelithiasis | 89 | 88914 | 27350 | 18165544 | 0.66 | -0.88 |
| SGLT2i | Biliary colic | 9 | 88994 | 2479 | 18190415 | 0.74 | -1.27 |
| SGLT2i | Bile duct stone | 8 | 88995 | 2704 | 18190190 | 0.6 | -1.6 |

**Table S4. The principles of disproportionate measurement and the criteria for signal detection.**

| **Method** | **Calculation formula** | **Criteria** |
| --- | --- | --- |
| **ROR** | $ROR=\frac{a / c}{b / d}$ | a ≥ 3  ROR ≥ 1  95%CI (lower limit) > 1 |
|  | $SE(lnROR)=\sqrt{\frac{1}{a}+\frac{1}{b}+\frac{1}{c}+\frac{1}{d}}$ |  |
|  | $95\%CI= e^{\ln\left( ROR \right)\pm1.96se}$ |  |
| **IC** | $IC = log₂[a(a+b+c+d)/((a+b)(a+c))]$ | IC_025_ >0 |
|  | $SE(IC) = 1/[ln(2) \times\surd a]$ |  |
|  | $IC₀₂₅ = IC - 1.96 \times SE(IC)$ |  |

**Table S5. Clinical and demographic characteristics of adverse events associated with Liraglutide, Semaglutide, Tirzepatide and Dulaglutide.**

| **Characteristic** | **Liraglutide**  **n = 33515** | **Semaglutide**  **n = 55319** | **Tirzepatide**  **n = 98677** | **Dulaglutide**  **n = 70592** |
| --- | --- | --- | --- | --- |
| **Gender** | | | | |
| Female | 63.1% | 61.9% | 62.4% | 51.7% |
| Male | 32.1% | 30.7% | 20.1% | 37.4% |
| **Age** | | | | |
| <18 | 0.2% | 0.2% | 0.0% | 0.1% |
| 18–44 | 8.7% | 8.3% | 18.8% | 3.5% |
| 45–64 | 33.8% | 22.2% | 35.9% | 18.7% |
| ≥65 | 21.3% | 21.0% | 15.6% | 17.3% |
| Unknown or missing | 36.0% | 48.3% | 29.7% | 60.4% |
| **Reporter type** | | | | |
| Consumer | 61.2% | 70.8% | 94.3% | 88.4% |
| Health professional | 11.8% | 8.9% | 3.1% | 5.4% |
| Pharmacist | 2.8% | 4.6% | 0.9% | 2.4% |
| Physician | 23.3% | 15.3% | 1.6% | 3.5% |
| Missing | 1.0% | 0.3% | 0.1% | 0.2% |
| **Report year** | | | | |
| 2010 | 2,652 (7.9%) | Not reported | Not reported | Not reported |
| 2011 | 5,261 (15.7%) | Not reported | Not reported | Not reported |
| 2012 | 3,643 (10.9%) | Not reported | Not reported | Not reported |
| 2013 | 1,784 (5.3%) | Not reported | Not reported | Not reported |
| 2014 | 1,143 (3.4%) | Not reported | Not reported | 9 (0.0%) |
| 2015 | 3,561 (10.6%) | Not reported | Not reported | 1,548 (2.2%) |
| 2016 | 3,080 (9.2%) | Not reported | Not reported | 3,480 (4.9%) |
| 2017 | 1,900 (5.7%) | Not reported | Not reported | 5,572 (7.9%) |
| 2018 | 2,518 (7.5%) | 1,043 (1.9%) | Not reported | 6,382 (9.0%) |
| 2019 | 1,324 (4.0%) | 1,308 (2.4%) | Not reported | 7,852 (11.1%) |
| 2020 | 1,398 (4.2%) | 2,879 (5.2%) | Not reported | 8,831 (12.5%) |
| 2021 | 1,152 (3.4%) | 3,743 (6.8%) | Not reported | 10,857 (15.4%) |
| 2022 | 1,011 (3.0%) | 5,583 (10.1%) | 3,113 (3.2%) | 9,246 (13.1%) |
| 2023 | 1,023 (3.1%) | 8,070 (14.6%) | 15,776 (16.0%) | 7,781 (11.0%) |
| 2024 | 996 (3.0%) | 13,435 (24.3%) | 37,106 (37.6%) | 5,436 (7.7%) |
| 2025 | 1,069 (3.2%) | 19,258 (34.8%) | 42,682 (43.3%) | 3,598 (5.1%) |

**Table S6. Distribution of AEs of GLP-1RAs in System Organ Class (SOC).**

| **System Organ Class (SOC)** | **Liraglutide**  **n = 85236** | **Semaglutide**  **n = 182495** | **Tirzepatide**  **n = 217837** | **Dulaglutide**  **n = 169902** |
| --- | --- | --- | --- | --- |
| Blood and lymphatic system disorders | 216 | 408 | 411 | 230 |
| Cardiac disorders | 1322 | 2199 | 1457 | 1604 |
| Congenital, familial and genetic disorders | 66 | 133 | 39 | 33 |
| Ear and labyrinth disorders | 215 | 650 | 543 | 730 |
| Endocrine disorders | 351 | 467 | 304 | 300 |
| Eye disorders | 1107 | 5044 | 2545 | 3361 |
| Gastrointestinal disorders | 26269 | 48215 | 44144 | 38099 |
| General disorders and administration site conditions | 11477 | 20947 | 54501 | 38278 |
| Hepatobiliary disorders | 1403 | 2242 | 1522 | 876 |
| Immune system disorders | 374 | 575 | 1287 | 943 |
| Infections and infestations | 1851 | 4473 | 3244 | 3305 |
| Injury, poisoning and procedural complications | 4080 | 23586 | 58989 | 25764 |
| Investigations | 9342 | 13754 | 11149 | 21245 |
| Metabolism and nutrition disorders | 4959 | 12283 | 7449 | 6868 |
| Musculoskeletal and connective tissue disorders | 2058 | 4858 | 3915 | 3473 |
| Neoplasms benign, malignant and unspecified (incl cysts and polyps) | 2695 | 1769 | 746 | 1541 |
| Nervous system disorders | 5657 | 12447 | 8628 | 8264 |
| Other/unknown | 1322 | 286 | 42 | 3042 |
| Pregnancy, puerperium and perinatal conditions | 157 | 289 | 146 | 34 |
| Product issues | 1018 | 3172 | 670 | 77 |
| Psychiatric disorders | 1914 | 6749 | 4997 | 3246 |
| Renal and urinary disorders | 1128 | 2326 | 1503 | 1675 |
| Reproductive system and breast disorders | 377 | 919 | 859 | 268 |
| Respiratory, thoracic and mediastinal disorders | 1629 | 3319 | 2495 | 2225 |
| Skin and subcutaneous tissue disorders | 2499 | 5259 | 4687 | 3287 |
| Social circumstances | 142 | 847 | 227 | 51 |
| Surgical and medical procedures | 880 | 3584 | 197 | 82 |
| Vascular disorders | 728 | 1695 | 1141 | 1001 |
| Total | 85236 | 182495 | 217837 | 169902 |

**Table S7. Clinical and demographic characteristics of biliary adverse events associated with Liraglutide, Semaglutide, Tirzepatide and Dulaglutide.**

| **Characteristic** | **Reports of biliary adverse events** | | | |
| --- | --- | --- | --- | --- |
|  | Liraglutide  n = 528, (%) | Semaglutide  n = 931, (%) | Tirzepatide  n = 562, (%) | Dulaglutide  n = 247, (%) |
| Sex |  |  |  |  |
| Female | 361 (68.4%) | 571 (61.3%) | 366 (65.1%) | 124 (50.2%) |
| Male | 147 (27.8%) | 319 (34.3%) | 119 (21.2%) | 108 (43.7%) |
| Age (year) |  |  |  |  |
| <18 | 7 (1.3%) | 5 (0.5%) | 0 (0.0%) | 0 (0.0%) |
| 18–44 | 128 (24.2%) | 185 (19.9%) | 148 (26.3%) | 25 (10.1%) |
| 45–64 | 181 (34.3%) | 320 (34.4%) | 174 (31.0%) | 69 (27.9%) |
| ≥65 | 103 (19.5%) | 211 (22.7%) | 54 (9.6%) | 78 (31.6%) |
| Unknown or missing | 109 (20.6%) | 210 (22.6%) | 186 (33.1%) | 75 (30.4%) |
| Weight (kg) |  |  |  |  |
| ≤80 | 59 (11.2%) | 125 (13.4%) | 44 (7.8%) | 15 (6.1%) |
| 80–100 | 106 (20.1%) | 167 (17.9%) | 77 (13.7%) | 22 (8.9%) |
| ≥100 | 126 (23.9%) | 186 (20.0%) | 60 (10.7%) | 40 (16.2%) |
| Unknown or missing | 237 (44.9%) | 453 (48.7%) | 381 (67.8%) | 170 (68.8%) |
| Reporter type |  |  |  |  |
| Consumer | 178 (33.7%) | 422 (45.3%) | 493 (87.7%) | 161 (65.2%) |
| Health Professional | 87 (16.5%) | 99 (10.6%) | 32 (5.7%) | 38 (15.4%) |
| Pharmacist | 34 (6.4%) | 68 (7.3%) | 13 (2.3%) | 19 (7.7%) |
| Physician | 224 (42.4%) | 333 (35.8%) | 22 (3.9%) | 28 (11.3%) |
| Outcome |  |  |  |  |
| Death | 9 (1.7%) | 12 (1.3%) | 8 (1.4%) | 4 (1.6%) |
| Disability | 4 (0.8%) | 23 (2.5%) | 18 (3.2%) | 3 (1.2%) |
| Hospitalization | 328 (62.1%) | 585 (62.8%) | 301 (53.6%) | 157 (63.6%) |
| Life-Threatening | 21 (4.0%) | 41 (4.4%) | 39 (6.9%) | 7 (2.8%) |

**Table S8. Disproportionality analysis associated with pancreatic adverse events at the PT level, with respect to study drugs.**

| **GLP-1RAs** | **PT** | ***N*** | **ROR** | **95%CI** | **IC** | **IC025-975** |
| --- | --- | --- | --- | --- | --- | --- |
|  |  |  |  |  |  |  |
| Liraglutide | Pancreatitis | 2224 | 31.57 | 30.21-32.99 | 4.81 | 4.76 |
|  | Pancreatitis acute | 640 | 19.63 | 18.13-21.26 | 4.22 | 4.11 |
|  | Pancreatitis necrotizing | 36 | 11.32 | 8.13-15.74 | 3.49 | 3.03 |
| Semaglutide | Pancreatitis | 1262 | 10.12 | 9.56-10.71 | 3.27 | 3.19 |
|  | Pancreatitis acute | 257 | 4.6 | 4.07-5.21 | 2.18 | 2.01 |
|  | Pancreatitis necrotizing | 75 | 14.6 | 11.58-18.4 | 3.82 | 3.5 |
| Tirzepatide | Pancreatitis | 868 | 3.8 | 3.55-4.07 | 1.9 | 1.8 |
|  | Pancreatitis acute | 205 | 2.04 | 1.78-2.34 | 1.02 | 0.83 |
|  | Pancreatitis necrotizing | 25 | 2.64 | 1.78-3.92 | 1.42 | 0.86 |
| Dulaglutide | Pancreatitis | 1331 | 8.34 | 7.89-8.81 | 3.0 | 2.92 |
|  | Pancreatitis acute | 198 | 2.76 | 2.4-3.18 | 1.46 | 1.26 |
|  | Pancreatitis necrotizing | 32 | 4.75 | 3.35-6.74 | 2.25 | 1.76 |

**Table S9. Disproportionality analysis associated with biliary adverse events at the PT level.**

| **GLP-1RAs** | **PT** | ***N*** | **ROR** | **95%CI** | **IC** | **IC025-975** |
| --- | --- | --- | --- | --- | --- | --- |
|  |  |  |  |  |  |  |
| Liraglutide | Cholelithiasis | 346 | 7.02 | 6.31-7.8 | 2.78 | 2.63 |
|  | Biliary colic | 47 | 10.5 | 7.86-14.01 | 3.38 | 2.97 |
|  | Cholecystitis | 91 | 4.98 | 4.05-6.12 | 2.31 | 2.02 |
|  | Bile duct stone | 32 | 6.51 | 4.59-9.22 | 2.71 | 2.22 |
|  | Cholecystitis infective | 18 | 4.11 | 2.59-6.54 | 2.07 | 1.42 |
|  | Cholecystitis chronic | 18 | 1.47 | 0.93-2.34 | 0.59 | -0.06 |
|  | Cholecystitis acute | 53 | 5.81 | 4.43-7.62 | 2.54 | 2.15 |
| Semaglutide | Cholelithiasis | 518 | 6.39 | 5.86-6.97 | 2.64 | 2.52 |
|  | Biliary colic | 111 | 15.41 | 12.74-18.65 | 3.89 | 3.63 |
|  | Cholecystitis | 253 | 8.53 | 7.53-9.67 | 3.06 | 2.88 |
|  | Bile duct stone | 76 | 9.51 | 7.57-11.95 | 3.22 | 2.9 |
|  | Cholecystitis infective | 40 | 5.58 | 4.08-7.63 | 2.48 | 2.04 |
|  | Cholecystitis chronic | 30 | 1.49 | 1.04-2.13 | 0.59 | 0.08 |
|  | Cholecystitis acute | 84 | 5.61 | 4.52-6.96 | 2.47 | 2.17 |
| Tirzepatide | Cholelithiasis | 378 | 2.58 | 2.33-2.86 | 1.35 | 1.21 |
|  | Biliary colic | 53 | 4.01 | 3.06-5.27 | 1.99 | 1.61 |
|  | Cholecystitis | 111 | 2.06 | 1.71-2.48 | 1.04 | 0.77 |
|  | Bile duct stone | 23 | 1.58 | 1.05-2.38 | 0.68 | 0.1 |
|  | Cholecystitis infective | 28 | 2.17 | 1.5-3.15 | 1.14 | 0.61 |
|  | Cholecystitis chronic | 2 | 0.06 | 0.01-0.22 | -3.85 | -5.64 |
|  | Cholecystitis acute | 51 | 1.89 | 1.43-2.49 | 0.93 | 0.53 |
| Dulaglutide | Cholelithiasis | 152 | 1.44 | 1.23-1.69 | 0.53 | 0.3 |
|  | Biliary colic | 15 | 1.56 | 0.94-2.6 | 0.69 | -0.03 |
|  | Cholecystitis | 51 | 1.31 | 1.0-1.73 | 0.41 | 0.01 |
|  | Bile duct stone | 15 | 1.43 | 0.86-2.38 | 0.57 | -0.15 |
|  | Cholecystitis infective | 11 | 1.19 | 0.66-2.15 | 0.31 | -0.52 |
|  | Cholecystitis acute | 26 | 1.34 | 0.91-1.97 | 0.45 | -0.1 |

**Table S10. Joint analysis of pancreatic-biliary co-reporting and the pancreatic signal after excluding co-reported biliary events.**

| **Drug** | **Co-reported pancreatic+biliary ICSRs, n (%)** | **Pancreatic ROR after excluding biliary co-reporting (95% CI)** | **IC025** |
| --- | --- | --- | --- |
| Liraglutide | 155 (5.4%) | 28.5 | 4.6 |
| Semaglutide | 118 (7.5%) | 8.49 | 2.95 |
| Tirzepatide | 98 (9.0%) | 3.16 | 1.54 |
| Dulaglutide | 62 (4.0%) | 6.77 | 2.63 |

**Table S11. Most common co-reported pancreatic and biliary Preferred Terms.**

| **Drug** | **Most common pancreatic+biliary PT combinations** | **n** |
| --- | --- | --- |
| Liraglutide | Pancreatitis + cholelithiasis | 89 |
| Liraglutide | Pancreatitis acute + cholelithiasis | 28 |
| Semaglutide | Pancreatitis + cholelithiasis | 65 |
| Semaglutide | Pancreatitis + cholecystitis | 30 |
| Tirzepatide | Pancreatitis + cholelithiasis | 50 |
| Tirzepatide | Pancreatitis acute + cholelithiasis | 33 |
| Dulaglutide | Pancreatitis + cholelithiasis | 30 |
| Dulaglutide | Pancreatitis acute + cholelithiasis | 16 |

**Table S12. Sensitivity analysis of study drugs using narrow-component MedDRA Standardised MedDRA Queries (SMQs) and cholecystectomy, with underlying two-by-two contingency table counts.**

| **Drug** | **SMQ definition** | **a** | **b** | **c** | **d** | **ROR** | **IC025** |
| --- | --- | --- | --- | --- | --- | --- | --- |
| Liraglutide | SMQ Acute pancreatitis | 2906 | 30609 | 61665 | 18186717 | 28.00 | 4.57 |
| Liraglutide | Gallstone-related disorders | 376 | 33139 | 29453 | 18218929 | 7.02 | 2.64 |
| Liraglutide | Cholecystectomy | 90 | 33425 | 9760 | 18238622 | 5.03 | 2.03 |
| Semaglutide | SMQ Acute pancreatitis | 1594 | 53725 | 62977 | 18163601 | 8.56 | 2.96 |
| Semaglutide | Gallstone-related disorders | 589 | 54730 | 29240 | 18197338 | 6.70 | 2.59 |
| Semaglutide | Cholecystectomy | 210 | 55109 | 9640 | 18216938 | 7.20 | 2.63 |
| Tirzepatide | SMQ Acute pancreatitis | 1100 | 97577 | 63471 | 18119749 | 3.22 | 1.57 |
| Tirzepatide | Gallstone-related disorders | 395 | 98282 | 29434 | 18153786 | 2.48 | 1.16 |
| Tirzepatide | Cholecystectomy | 2 | 98675 | 9848 | 18173372 | 0.04 | -6.20 |
| Dulaglutide | SMQ Acute pancreatitis | 1567 | 69025 | 63004 | 18148301 | 6.54 | 2.58 |
| Dulaglutide | Gallstone-related disorders | 167 | 70425 | 29662 | 18181643 | 1.45 | 0.32 |
| Dulaglutide | Cholecystectomy | 4 | 70588 | 9846 | 18201459 | 0.10 | -4.41 |
| Metformin | SMQ Acute pancreatitis | 997 | 66291 | 63574 | 18151035 | 4.29 | 1.98 |
| Metformin | Gallstone-related disorders | 135 | 67153 | 29694 | 18184915 | 1.23 | 0.06 |
| Metformin | Cholecystectomy | 15 | 67273 | 9835 | 18204774 | 0.41 | -1.94 |
| SGLT-2 inhibitors | SMQ Acute pancreatitis | 1161 | 87842 | 63410 | 18129484 | 3.78 | 1.80 |
| SGLT-2 inhibitors | Gallstone-related disorders | 96 | 88907 | 29733 | 18163161 | 0.66 | -0.88 |
| SGLT-2 inhibitors | Cholecystectomy | 25 | 88978 | 9825 | 18183069 | 0.52 | -1.47 |
